# Population Pharmacodynamic Modeling and Simulation of the Respiratory Effect of Acetazolamide in Decompensated COPD Patients

**DOI:** 10.1371/journal.pone.0086313

**Published:** 2014-01-17

**Authors:** Nicholas Heming, Saïk Urien, Virginie Fulda, Ferhat Meziani, Arnaud Gacouin, Marc Clavel, Benjamin Planquette, Christophe Faisy

**Affiliations:** 1 Service de Réanimation Médicale, Hôpital Européen Georges Pompidou, Université Paris Descartes, Sorbonne Paris Cité, Assistance Publique−Hôpitaux de Paris, Paris, France; 2 CIC-01109 Cochin-Necker INSERM, Unité de Recherche Clinique, Hôpital Tarnier, Université Paris Descartes, Sorbonne Paris Cité, Assistance Publique−Hôpitaux de Paris, Paris, France; 3 Centre Régional de Pharmacovigilance, Hôpital Européen Georges Pompidou, Université Paris Descartes, Sorbonne Paris Cité, Assistance Publique−Hôpitaux de Paris, Paris, France; 4 Service de Réanimation Médicale, Hôpital Civil, Strasbourg, France; 5 Service de Réanimation Médicale et Infectieuse, Hôpital Pontchaillou, Rennes, France; 6 Service de Réanimation Polyvalente, CHU de Limoges, Limoges, France; 7 Service de Réanimation Médico-Chirurgicale, Hôpital André Mignot, Le Chesnay, France; New York University School of Medicine, United States of America

## Abstract

**Background:**

Chronic obstructive pulmonary disease (COPD) patients may develop metabolic alkalosis during weaning from mechanical ventilation. Acetazolamide is one of the treatments used to reverse metabolic alkalosis.

**Methods:**

619 time-respiratory (minute ventilation, tidal volume and respiratory rate) and 207 time-PaCO_2_ observations were obtained from 68 invasively ventilated COPD patients. We modeled respiratory responses to acetazolamide in mechanically ventilated COPD patients and then simulated the effect of increased amounts of the drug.

**Results:**

The effect of acetazolamide on minute ventilation and PaCO_2_ levels was analyzed using a nonlinear mixed effect model. The effect of different ventilatory modes was assessed on the model. Only slightly increased minute ventilation without decreased PaCO_2_ levels were observed in response to 250 to 500 mg of acetazolamide administered twice daily. Simulations indicated that higher acetazolamide dosage (>1000 mg daily) was required to significantly increase minute ventilation (*P*<.001 vs pre-acetazolamide administration). Based on our model, 1000 mg per day of acetazolamide would increase minute ventilation by >0.75 L min^−1^ in 60% of the population. The model also predicts that 45% of patients would have a decrease of PaCO2>5 mmHg with doses of 1000 mg per day.

**Conclusions:**

Simulations suggest that COPD patients might benefit from the respiratory stimulant effect after the administration of higher doses of acetazolamide.

## Introduction

Acetazolamide is a non selective carbonic anhydrase (CA) inhibitor. Carbonic anhydrases are key enzymes implicated in the homeostasis of the acid-base equilibrium. CA inhibitors induce metabolic acidosis by blocking isoforms of the enzyme located in the kidney [1], [2]. Acetazolamide is commonly used in the intensive care unit (ICU) for reversing metabolic alkalosis [3]. The drug has an excellent bioavailability, a relatively short half life and is eliminated unmetabolized from the plasma by the kidney.

Chronic obstructive pulmonary disease (COPD) is a leading cause of morbidity and mortality worldwide. The disease is marked by the occurrence of exacerbations affecting patient prognosis [4]. COPD decompensation is a frequent cause of intensive care unit (ICU) hospitalization [5], [6]. Severe exacerbations might lead to the need to establish mechanical invasive ventilation. Additionally, COPD is an established risk factor for developing postoperative pulmonary complications [7], [8]. Invasively ventilated COPD patients are at high risk of prolonged mechanical ventilation and persistent weaning failure [9]. Prolonged invasive mechanical ventilation is in turn associated with increased hospital mortality [10], [11]. Different modes of ventilator setting are used; volume targeted ventilation, which guarantees the administration of a determined tidal volume and pressure targeted ventilation which controls the pressure inside the airways. Neither method has been demonstrated as being superior for obtaining a faster weaning [12]. Acetazolamide appears to increase respiratory drive and might be beneficial in obtaining a faster weaning from mechanical ventilation, especially in COPD patients who have developed metabolic alkalosis. Indeed central drive might be reduced due to metabolic alkalosis, mechanical ventilation by itself and sedative or hypnotic medications [13].

However, the optimal dosage of acetazolamide to be administered to alkalotic COPD patients is unclear [3], [14]. We previously modeled the effect of acetazolamide on serum bicarbonate [15]. Our present aim was to assess the effect of acetazolamide on minute ventilation and PaCO_2_ levels in the same cohort of alkalotic mechanically ventilated COPD patients. Adequate levels of both respiratory rate and tidal volume are required in order to maintain PaCO_2_ levels during spontaneous breathing. We subsequently modeled the effect of acetazolamide on minute ventilation and PaCO_2_ and then, using Markov Chain Monte Carlo computations, simulated the effect of higher doses of acetazolamide on these respiratory parameters.

## Methods

### Patients and Study Design

Patients included in the cohort were hospitalised between October 2000 and October 2010 in the 18 bed medical ICU of a teaching hospital. This cohort has been previously described [15]. Due to the observational design, patient consent was waived, in accordance with French bioethics laws. The use of computerized medical data with protection of patient confidentiality was approved by the Commission Nationale de l’Informatique et des Libertés. Data were extracted from the files of 68 consecutive COPD patients. These patients were all treated by acetazolamide for the reversal metabolic alkalosis during the weaning period. COPD was diagnosed according to the Global Initiative for Chronic Obstructive Lung Disease (GOLD) criteria [16]. Readiness to wean was defined according to the criteria of the Sixth International Conference Consensus in Intensive Care Medicine held in 2005 [13]. Briefly, acetazolamide 250 to 500 mg was delivered over a couple of minutes intravenously twice a day. The weaning procedure from mechanical ventilation and the procedure for extubation follow a written protocol in our ICU. The weaning strategy (1) consisted of a progressive decrease in pressure support ventilation or volume-assisted ventilation with the use of progressively increased time on a T-piece and (2) was tailored by physicians in charge according to the difficulty of the weaning process [15].

### Variables

Baseline characteristics of the included patients at ICU admission were collected. Arterial blood gases realized during mechanical ventilation, before and up to 24 hours after administration of acetazolamide were recorded. Throughout acetazolamide treatment, data regarding respiratory parameters (mode of mechanical ventilation, respiratory rate, tidal volume and minute ventilation) were collected from patient’s charts. We also retrieved covariates identified as influencing acetazolamide pharmakodynamics as well as the length of mechanical ventilation, the length of stay in the ICU and outcome in the ICU.

### Pharmacodynamic Modeling

The pharmacokinetics of the drug was ascribed to a one-compartment open model [17], [18]. Elimination of acetazolamide was deemed to be linear, with an elimination half-life set at six hours [18]. The effect of acetazolamide on serum bicarbonate concentration (Bicar(t), in mmol l^−1^) has previously been modelized [15]. The influence of each covariate, which, based on our current understanding could influence the effect of the drug (age, gender, body weight, FEV_1_, FEV_1_/FVC, SAPSII, length of stay in the ICU, ICU mortality, serum creatinine, protide, chloride and potassium, administration of corticosteroid, of furosemide, of β_2_ agonists, type of ventilator mode) was tested on the model. Covariates were incorporated into the model if they improved the fit of the model to the data. The effect of acetazolamide on minute ventilation (MV(t), in l min^−1^) was related to Bicar(t) and then PaCO_2_ (PaCO2(t), in mmHg) was related to MV(t) as follows:
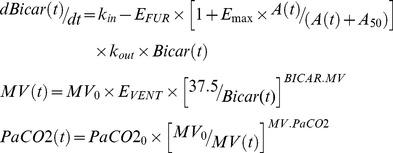
k_in_ (mmol l^−1 ^day^−1^) stands for the bicarbonate rate formation. k_out_ (day^−1^) is the first-order constant rate of bicarbonate elimination. k_in_ is deduced from the basal level plus the k_out_ parameters, i.e., at equilibrium (t = 0) the differential equation is zero and k_in_ = k_out_ x Bicar_0_. E_FUR_ stands for the effect of furosemide administration on bicarbonate equilibrium [15]. E_VENT_ stands for the effect of the mode of ventilation on PaCO_2_ equilibrium. A_50_ stands for the amount of drug that induces 50% of the maximal effect (E_max_). BICAR.MV and MV.PaCO2 are the power parameters for the effect of Bicar(t) on MV(t) and the effect of MV(t) on PaCO2(t).

### Data Analysis

Methods for data analysis have already been reported [15]. Data were analyzed using the nonlinear mixed-effect modeling software program Monolix version 3.1S Release 2 (http://wfn.software.monolix.org/). Parameters were estimated by computing the maximum likelihood estimation of the parameters without any approximation of the model (that is, no linearization) using the stochastic approximation expectation maximization algorithm combined with a Markov chain Monte Carlo procedure. A proportional error model was used to describe residual variability, and between-subject variability (BSV, or η) was ascribed to an exponential error model. Parameter shrinkage was calculated as [1 - sd(η)/ω], where sd(η) and ω are the standard deviation of individual η parameters and the population model estimate of the BSV, respectively. The likelihood ratio test, including the log-likelihood and the Akaike information criterion (AIC) were used to test different hypotheses regarding the final model, the covariate effects on pharmacokinetic parameters, the residual variability model (proportional versus proportional plus additive error model) and the structure of the variance-covariance matrix for the BSV parameters. Residuals were presented as normalized prediction distribution errors (NPDEs), based on the estimates of unbiased means and variances of the predictions by using 500 Monte Carlo simulations of the final model (the calculation includes a decorrelation step of the prediction errors). The mean values and variance of these normalized residues must not be different from 0 and 1, respectively. Diagnostic graphics and other statistics were derived using the R software program [19]. The results are expressed as numbers (%), means ± SD, or medians (range) for data with non-normal distributions. *P*<.05 was considered significant.

## Results

### Patients and Observations

Among the 68 investigated patients, a total of 619 time-respiratory (minute ventilation, respiratory rate and tidal volume) and 207 time-serum HCO_3_^−^ and PaCO_2_ observations were available for analysis with [median (range)] observations per patient: minute ventilation  = 9 (2–14); serum bicarbonates  = 3 (1–6); PaCO_2_ = 3 (1–6). The [mean ± SD] values of minute ventilation, tidal volume and respiratory rate were respectively 9.9±3.4 l min^−1^, 460±110 ml and 23±9 cycle min^−1^. The [mean (range)] length of mechanical ventilation was 18 (3–110) days. No serious side effects related to acetazolamide administration were recorded.

### Effect of Low Acetazolamide Dosage on Respiratory Parameters

**Figure 1:**
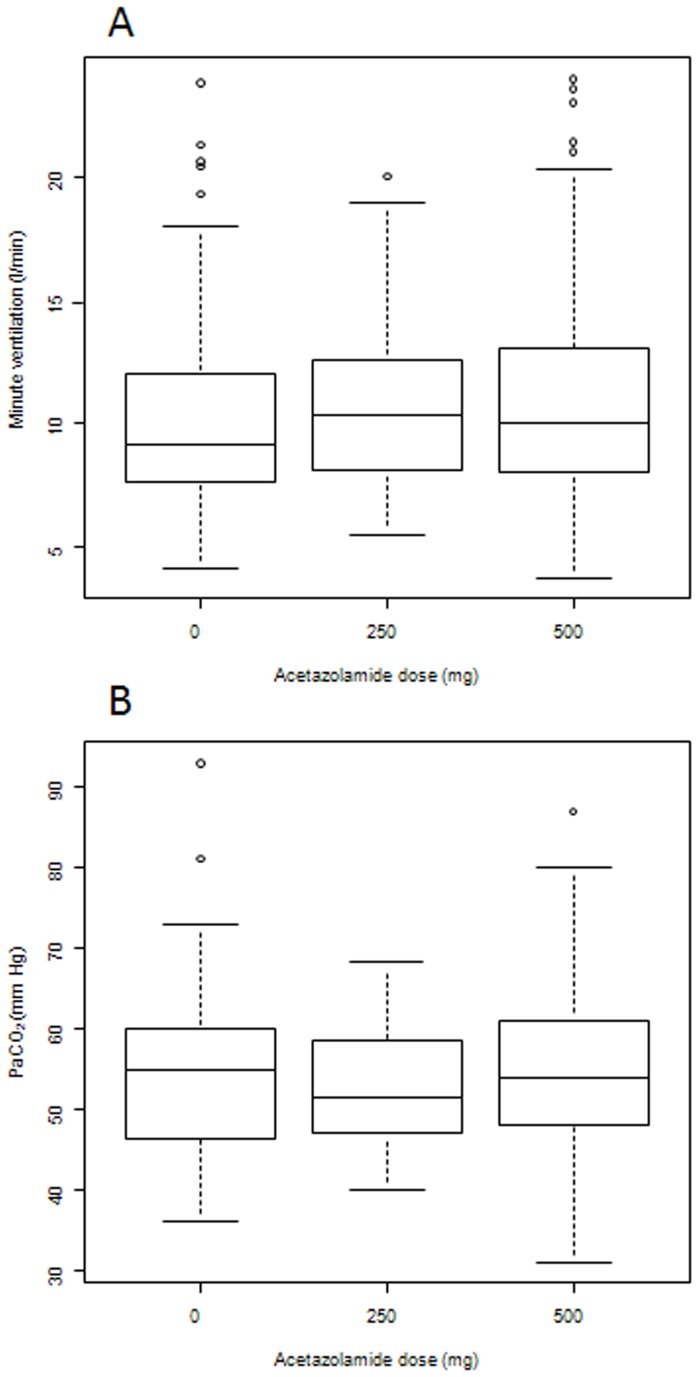
Difference between respiratory parameters pre-acetazolamide dose and at 24 hours. Differences between pre-acetazolamide dose minute ventilation and minute ventilation at 24 hours (A) and between pre-acetazolamide dose PaCO_2_ level and PaCO_2_ level at 24 hours (B) in 68 COPD patients, plotted according to the total quantity of acetazolamide administered daily. Boxplots show the median values, first and third quartiles and 10th and 90th percentiles. All values are observed. Usually administered doses of acetazolamide (250–500 mg) do not have a clinically relevant effect either on minute ventilation or on PaCO_2_ levels.

Firstly, we plotted the observed values of minute ventilation and PaCO_2_ according to the doses of acetazolamide administered twice daily (Fig. 1), confirming the moderate effect of the drug at usually administered doses. We also report the values of minute ventilation and of PaCO_2_ of all patients immediately before the administration of acetazolamide. As shown in Figure 1, 250 to 500 mg of acetazolamide administrated daily slightly increased minute ventilation, without reaching statistical significance (Fig. 1A) without decreasing PaCO_2_ levels (Fig. 1B).

**Figure 2:**
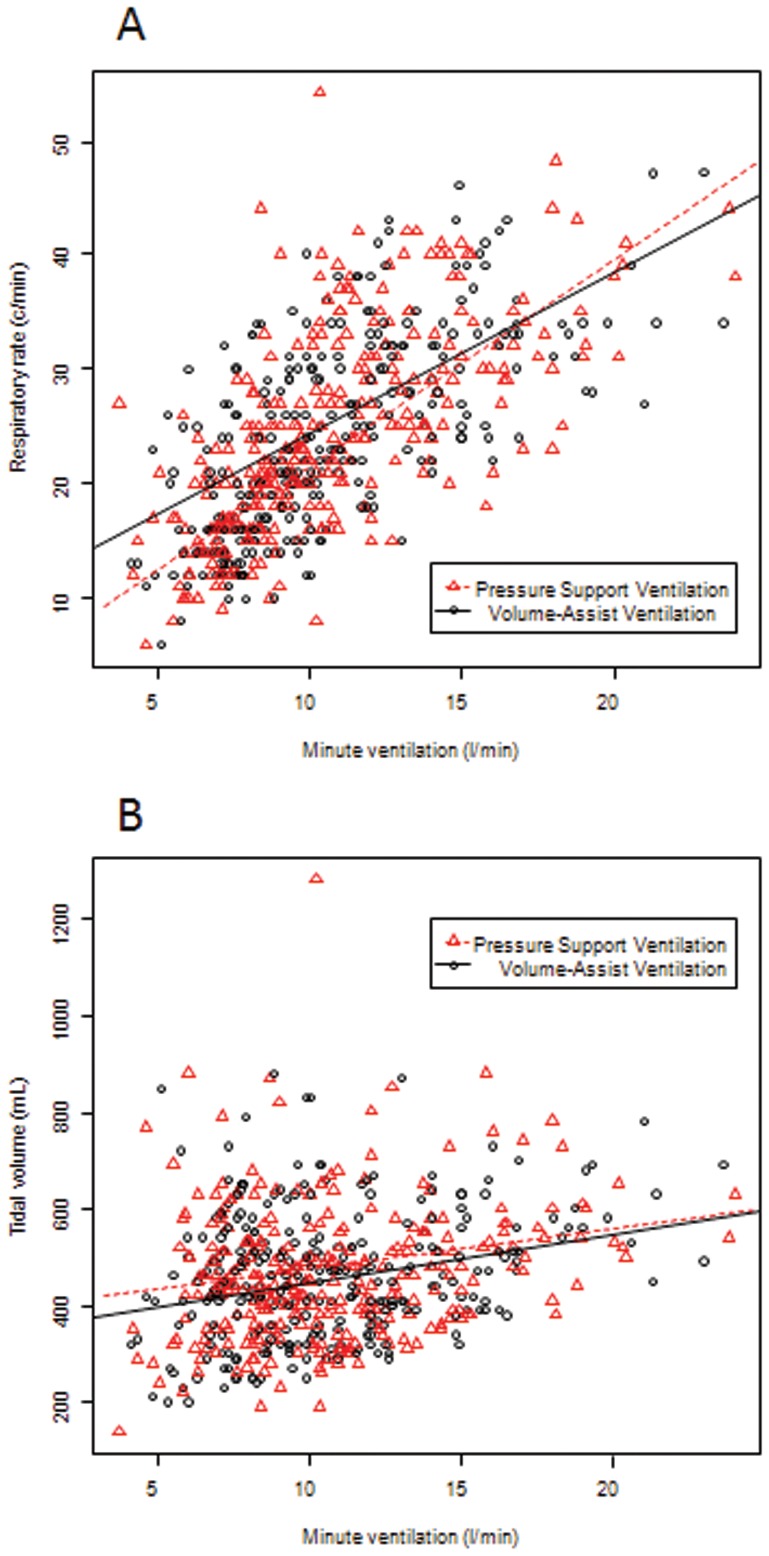
Relationship between minute ventilation and respiratory rate or tidal volume after treatment by acetazolamide. Relationship between minute ventilation and respiratory rate (A) or tidal volume (B) according to the ventilator mode in 68 COPD patients after treatment by acetazolamide (acetazolamide, 250 to 500 mg twice daily) during the weaning from mechanical ventilation period. The increase in minute ventilation after administration of acetazolamide was a consequence of the increase of respiratory rate rather than the increase of tidal volume, whatever the ventilator mode. Every point is one observation after acetazolamide administration (see text for details). Regression lines for both pressure-support (dotted line) and volume-assist (solid line) ventilation are represented on the figure.

We thereafter assessed whether respiratory rate or tidal volume played a greater role in determining the value of minute ventilation, depending on the mode of mechanical ventilation. We plotted values of minute ventilation after administration of acetazolamide in relation to tidal volume and respiratory rate and compared correlation coefficients. When minute ventilation was considered in relation to the respiratory rate, the amount of minute ventilation explained by the respiratory rate was higher in patients ventilated by volume-assist ventilation than those ventilated by pressure support ventilation (correlation coefficients and 95% confidence intervals, respectively 0.76 [0.70–0.815] and 0.60 [0.52–0.66] Fig. 2A). Conversely, the amount of minute ventilation explained by the tidal volume was similar in volume-assist ventilated patients and in pressure support ventilated patients (correlation coefficients and 95% confidence intervals, respectively 0.22 [0.09–0.35] and 0.24 [0.16–0.36] Fig. 2B).

### Effect of High Acetazolamide Dosage on Respiratory Parameters-pharmacodynamic Modeling and Subsequent Simulations

**Figure 3:**
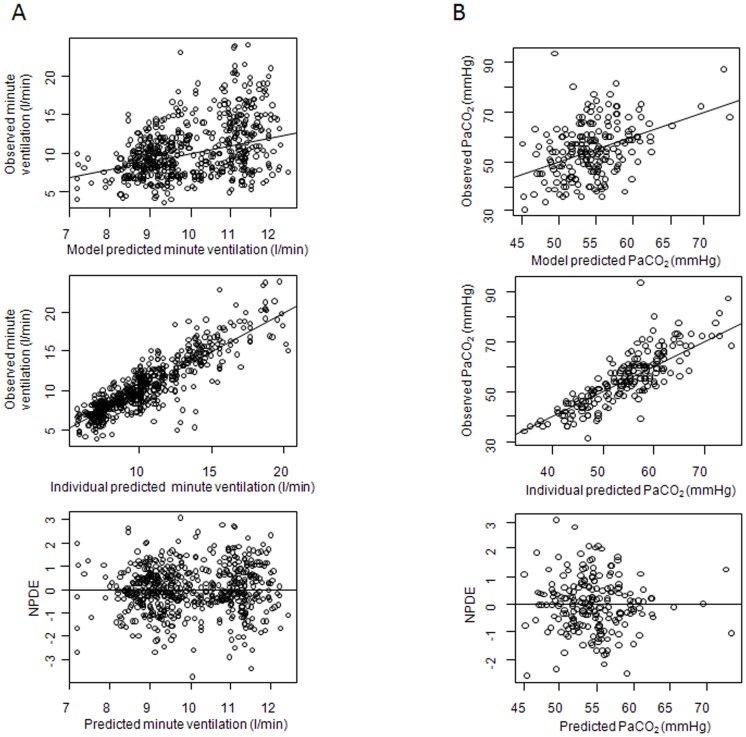
Goodness-of-fit plots for the final model of pharmakodynamics. Observed versus model-predicted minute ventilation (A) and observed versus model-predicted PaCO_2_ (B) for mean and individual predictions, and normalized prediction distribution errors (npde) versus predicted minute ventilation and PaCO_2_. The regression line is represented by the solid line. The mean and variance of the npde distribution were not significantly different from respectively 0 and 1 (*P*  = 0.63 and *P*  = 0.56, respectively for PaCO_2_ and *P*  = 0.59 and *P*  = 0.48 for minute ventilation; Wilcoxon signed-rank test and Fisher variance test, respectively) and from normality, illustrating robustness of minute ventilation and PaCO_2_ prediction after acetazolamide administration.

**Figure 4:**
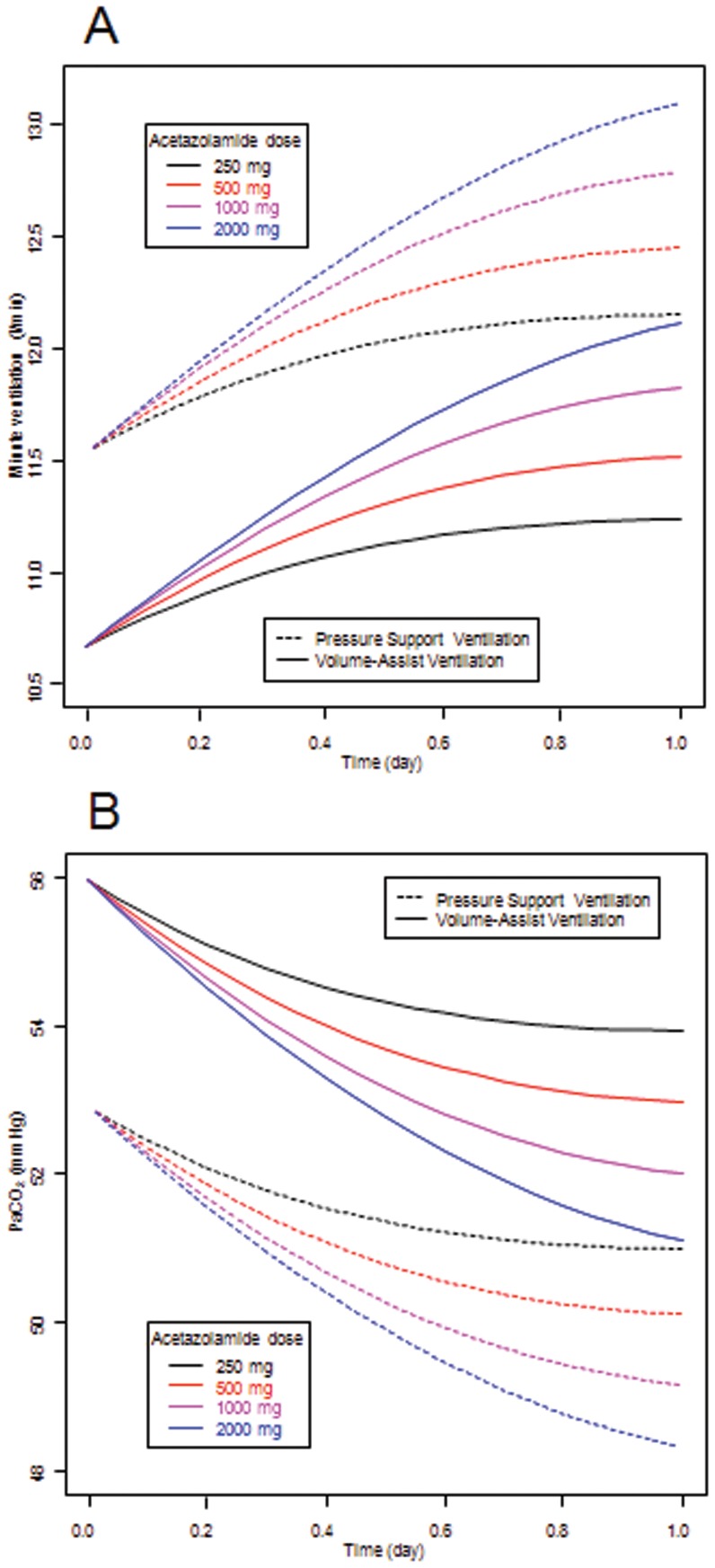
Model-predicted effect of once a day administration of 250, 500, 1000 or 2000 mg of acetazolamide. Predicted effect of the drug on minute ventilation (A) and PaCO_2_ (B) in patients ventilated either by pressure support ventilation or by volume assist ventilation. Modelization of acetazolamide pharmacodynamics was derived from 68 COPD patients with metabolic alkalosis during the weaning period. The model predicts that higher acetazolamide dosage (>1000 mg) is required to significantly increase minute ventilation or to decrease PaCO_2_ whatever the ventilator mode used.

**Figure 5:**
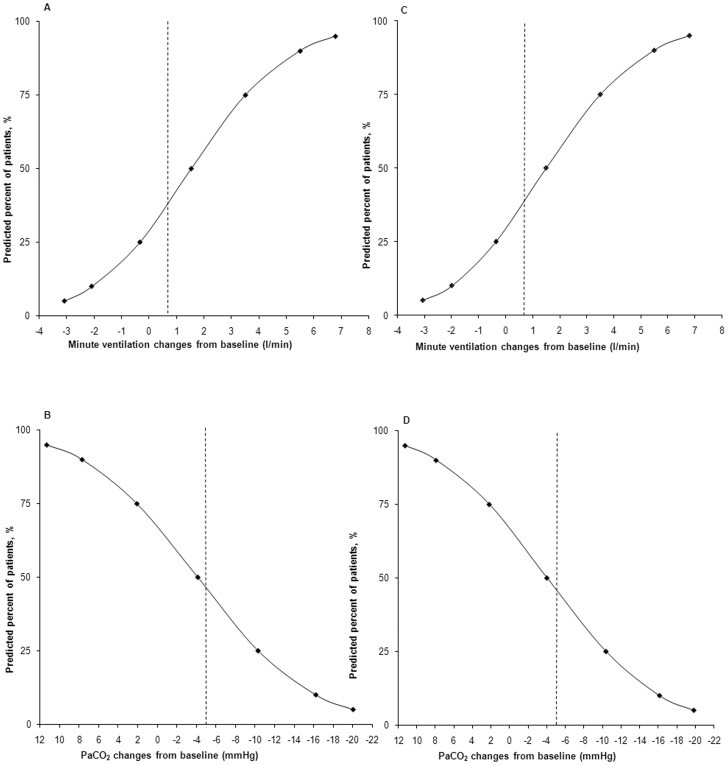
Model-predicted effect of once a day administration of 1000 mg of acetazolamide. Predicted effect of the drug on minute ventilation (A,C) and PaCO_2_ (B,D). Figures A and C predict the effect in patients supported by pressure support ventilation whereas figures B and D predict the effect in patients supported by volume assist-ventilation. Minute ventilation is predicted to increase by more than 0.75 l min^−1^ in 65% of patients (A) and PaCO_2_ is predicted to decrease by 5 mmHg in 45% of patients (B) supported by pressure support ventilation treated by 1000 mg day^−1^ of acetazolamide. Minute ventilation is predicted to increase by more than 0.75 l min^−1^ in 60% of patients (C) and PaCO_2_ is predicted to decrease by 5 mmHg in 45% of patients (D) supported by volume-assist ventilation treated by 1000 mg day^−1^ of acetazolamide. Vertical dotted lines have been added at clinically relevant values of minute ventilation of 0.75 l min^−1^ and PaCO_2_ of 5 mmHg.

Since the effect of acetazolamide on minute ventilation at usually administered doses was modest and in order to determine whether higher doses of acetazolamide might have more of an effect, we subsequently modeled the effect of acetazolamide on minute ventilation and on PaCO_2_ levels during pressure-support or volume-assist ventilation. We chose the model that had the lowest AIC value than the next best fitting model. The final model equations were:
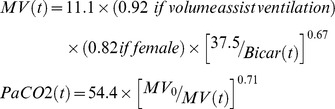



Using this model, we simulated the effect of higher doses of acetazolamide on minute ventilation and on PaCO_2_ levels during pressure-support or volume-assist ventilation. We were unable to determine the E_max_ and the A_50_ parameters at the same time. We therefore set the value of E_max_ at 1. Table 1 summarizes the estimates of the parameters of the model. Figure 3 illustrates the goodness of fit of the model. Model errors over time were plotted for each subject without any systematic deviation of error over time being obvious (data not shown). According to model simulations, a higher dosage of acetazolamide (>1000 mg daily) is required to significantly increase minute ventilation (Fig. 4A). Our model predicted that minute ventilation increase is more important (by 10%) during pressure-support than during volume-assist ventilation (Fig. 4A). However, PaCO_2_ levels evolved similarly when acetazolamide dosage was increased during pressure-support and volume-assist ventilation (Fig. 4B). We subsequently estimated the proportion of patients who would benefit from the daily administration of 1000 mg of acetazolamide (Fig. 5). Based on our model, doses of 1000 mg day^−1^ would increase minute ventilation by at least 0.75 L min^−1^ in 65% of patients (Fig. 5A) and PaCO_2_ would decrease by 5 mmHg in 45% of patients (Fig. 5B) supported by pressure support ventilation. Likewise, minute ventilation is predicted to increase by more than 0.75 l min^−1^ in 60% of patients (Fig. 5C) and PaCO_2_ is predicted to decrease by 5 mmHg in 45% of patients (Fig. 5D) supported by volume-assist ventilation.

**Table 1:** Parameter estimates of the final population model.

Parameter	Estimate (%rse)	BSV (%rse)
Half life, day	0.25 (fixed)	NA
E_max_	1 (fixed)	NA
Bicar_0_, mmol l^−1^	35.4 (2)	0.10 (9)
× by SAPS effect, [SAPS II/50]^−0.11^	−0.11 (41)	NA
× by corticosteroid effect, if present	1.10 (40)	NA
× by serum chloride effect, [Chloride/100]^−1.17^	−1.17 (18)	NA
*k_out_*, day^−1^	0.314 (20)	0.995 (12)
A_50_	163 (27)	NA
Furosemide_50_	204	NA
MV_0_, l min^−1^	11.1 (5)	0.225 (59)
× by volume-assist ventilation effect, if present	0.92 (2)	NA
× by 0.82 if gender = female	0.82 (30)	NA
× by serum bicarbonate effect, [37.5/Bicar(t)]^0.67^	0.67 (14)	NA
PaCO2_0_, mmHg	54.4 (2)	0.102 (15)
× by minute ventilation effect, [MV_0_/MV]^0.71^	0.71 (21)	0.56 (36)
Residual variability, proportional model; Bicar(t)	0.044 (8)	NA
Residual variability, proportional model; MV(t)	0.16 (3)	NA
Residual variability, proportional model; PaCO2(t)	0.12 (6)	NA

*Definition of abbreviations:* E_max_ = maximal effect of the drug; A_50_ =  amount of acetazolamide that instantaneously induces 50% of putative maximal effect on serum bicarbonate; Bicar_0_ =  bicarbonate baseline level; MV_0_ =  minute ventilation at baseline level; PaCO2_0_ =  PaCO2 at baseline level; BSV = between-subject variability; *k_out_* = first-order constant rate for acetazolamide effect kinetics; %rse = percent relative standard error; SAPS II = simplified acute physiology score II at intensive care unit admission; NA = not applicable; Furosemide_50_ =  amount of furosemide that instantaneously induces 50% of putative maximal effect on serum bicarbonate.

## Discussion

This study is a continuation of our previous published work [15]. The results of the present study suggest that higher doses of acetazolamide are necessary to induce a substantial increase in minute ventilation and hence a decrease in PaCO_2_ in alkalotic mechanically ventilated COPD patients. Additionally, this study also suggests that the increase in minute ventilation following the administration of acetazolamide is obtained at the price of an increase in respiratory rate rather than tidal volume, whatever the ventilatory mode. Finally, our findings suggest that acetazolamide seems to have more of an effect in COPD patients under pressure-support ventilation as opposed to volume-assist ventilation. We undertook this study because acetazolamide has little effect at usually administered doses.

Respiratory acidosis and metabolic alkalosis are the two main acid-base disturbances found in COPD patients during respiratory decompensations. Metabolic alkalosis results when the H^+^ concentration in the extracellular compartment is decreased. Persistent metabolic alkalosis after normalization of PaCO_2_ is frequent in these patients [20], [21]. Mixed acid-base balance disturbances (the association of chronic respiratory acidosis and metabolic alkalosis) can also be observed. Indeed, in mechanically ventilated COPD patients during the weaning period, the most frequently observed acid-base disorders are mixed, characterized by elevated serum bicarbonate and variable pH levels [14], [15].

It has previously been reported that up to 14% of all patients hospitalized in an intensive care unit had received acetazolamide during their stay [3]. Surprisingly no randomized trial has yet assessed the effect of acetazolamide on survival or length of mechanical ventilation in decompensated COPD patients. Acetazolamide administered to COPD patients at the usual dose during the weaning process induces only a small decrease in serum HCO_3_^−^ concentration [14]. Such a modest effect could be in part responsible for the lack of a consistent effect of acetazolamide on minute ventilation in these patients.

Acetazolamide inhibits the CA enzyme, mainly in the kidney and red blood cells and induces metabolic acidosis [22]. Metabolic acidosis stimulates peripheral and central chemoreceptors, increasing both minute ventilation and PaO_2_ [23], [24]. Acetazolamide may allow COPD patients suffering from metabolic alkalosis to be weaned more rapidly from mechanical ventilation. Regarding the mechanism by which minute ventilation is increased, our findings suggest the involvement of respiratory rate rather than tidal volume, at least in the pressure support ventilator mode. However, by raising respiratory rate, acetazolamide may increase the work of breathing, potentially worsening the clinical condition of these patients [25]. Indeed, skeletal muscle atrophy already occurs in patients suffering from advanced COPD and mechanical ventilation by itself might induce atrophy of the diaphragm [26], [27]. Additionally, in an animal model lower doses of acetazolamide negatively affected diaphragmatic strength [28]. Lastly and worryingly, the increase in respiratory rate in COPD patients could decrease expiratory time thus enhancing intrinsic positive end-expiratory pressure [29].

The tissue compartmentalization of CA isoforms and the low selectivity of acetazolamide may explain, in part, the complexity of the effect of the drug on the ventilatory drive in COPD patients [22], [30].

There is little evidence to support only one dosing strategy of acetazolamide in mechanically ventilated patients. Mazur et al. suggested that a single daily dose of 500 mg of acetazolamide reverses metabolic alkalosis as effectively as multiple doses of 250 mg [3]. However, acetazolamide administration at a daily dose of 500 mg only moderately reduces serum HCO_3_^−^ levels [14].

The administration of acetazolamide improves arterial blood gas parameters without significantly changing minute ventilation in spontaneously breathing COPD patients [31]. Our results partly confirm these data regarding the presence of a dose-response effect on respiratory parameters. On the other hand, Swenson et al. [22] and Teppema et al. [30] showed that acetazolamide increases minute ventilation by reducing excess base levels in the healthy subject. Herein we found that minute ventilation is expected to intensify when COPD patients are under pressure-support ventilation. Future investigations are needed to determine how acetazolamide improves minute ventilation according to the level of pressure-support.

We sought to determine the effect of various covariates on some of the main physiological respiratory variables. A pharmacodynamic model seemed the best option for such a task. Additionally, acetazolamide was only administered to patients during the weaning from mechanical ventilation period, which was undertaken following a written protocol, meaning that patients were in a relatively steady state. The present investigation was undertaken to assess the effect of several acetazolamide dosages on physiological respiratory parameters. We did not seek to assess the effect of acetazolamide on relevant clinical endpoints.

This study has several limitations, including the retrospective observational design. Patients of the cohort were issued from only one center. Our population was nevertheless similar to COPD patients treated by invasive mechanical ventilation [32]. We made a number of assumptions in this work. For instance, regarding the linear elimination of the drug, we based our assumption on previously published results and on the fact that a vast majority of drugs follow such a rule. The results of our simulations regarding the use of increased doses of acetazolamide are being prospectively confirmed. Indeed, a large randomized controlled trial (NCT01627639) assessing the effect of higher doses of acetazolamide is currently underway.

## Conclusion

In summary, our findings suggest that mechanically ventilated COPD patients would require more than 1000 mg of acetazolamide a day in order to benefit from the respiratory stimulant effect of the drug during the weaning period. Such an increase in acetazolamide dosage would induce an increase in respiratory rate rather than tidal volume in COPD patients. A large randomized trial using higher acetazolamide dosage is needed to demonstrate the benefit of this drug during the weaning process of mechanically ventilated COPD patients.
